# A Stacked Generalization Model to Enhance Prediction of Earthquake-Induced Soil Liquefaction

**DOI:** 10.3390/s22197292

**Published:** 2022-09-26

**Authors:** Sri Preethaa, Yuvaraj Natarajan, Arun Pandian Rathinakumar, Dong-Eun Lee, Young Choi, Young-Jun Park, Chang-Yong Yi

**Affiliations:** 1 Department of Artificial Intelligence and Data Science, KPR Institute of Engineering and Technology, Coimbatore 641665, India; 2 Intelligent Construction Automation Center, Kyungpook National University, 80, Daehak-ro, Buk-gu, Daegu 41566, Korea; 3 Research Engineer, QpiCloud Technologies, Bangalore 560045, India; 4 School of Architecture, Civil, Environment and Energy Engineering, Kyungpook National University, 80 Daehak-ro, Buk-gu, Daegu 41566, Korea; 5 Earth Turbine, 36, Dongdeok-ro 40-gil, Jung-gu, Daegu 41905, Korea

**Keywords:** liquefaction, prediction, machine learning, ensemble models, settlement, data augmentation

## Abstract

Earthquakes cause liquefaction, which disturbs the design phase during the building construction process. The potential of earthquake-induced liquefaction was estimated initially based on analytical and numerical methods. The conventional methods face problems in providing empirical formulations in the presence of uncertainties. Accordingly, machine learning (ML) algorithms were implemented to predict the liquefaction potential. Although the ML models perform well with the specific liquefaction dataset, they fail to produce accurate results when used on other datasets. This study proposes a stacked generalization model (SGM), constructed by aggregating algorithms with the best performances, such as the multilayer perceptron regressor (MLPR), support vector regression (SVR), and linear regressor, to build an efficient prediction model to estimate the potential of earthquake-induced liquefaction on settlements. The dataset from the Korean Geotechnical Information database system and the standard penetration test conducted on the 2016 Pohang earthquake in South Korea were used. The model performance was evaluated by using the R^2^ score, mean-square error (MSE), standard deviation, covariance, and root-MSE. Model validation was performed to compare the performance of the proposed SGM with SVR and MLPR models. The proposed SGM yielded the best performance compared with those of the other base models.

## 1. Introduction

On 15 November 2017, a 5.4 Mw earthquake affected the industrial city of Pohang, South Korea. The earthquake’s hypocenter was at a depth of 4 km, and this event ranked second to the 5.5 Mw Gyeongju earthquake, which occurred in South Korea in 2016 with a hypocenter at a depth of 14 km. Even though the hypocenter was at a low depth, the Pohang earthquake caused massive damages to buildings and other facilities. Ground deformations, such as cracks and soil blows, were found around the epicenter [1]. In general, an earthquake causes surface ruptures, surface shaking, landslides, tsunamis, and liquefaction when its magnitude is >6.5 Mw. Most of the damage that occurred in sedimentary basins was because of solid trembling and shuddering of soft sediments of the soil. Liquefaction occurs when the saturated silt or salty soil loses its stiffness and strength, thus transforming into a fluid state owing to the trembling and loading during the earthquake [2]. During the liquefaction phenomenon, water-pore pressure increases, causing the soil to lose its granularity; thus, the ground becomes feeble and unable to withstand the stresses of the structural load from its foundations. Eventually, the structures sink and cause instability, such as leaning or the collapse of buildings [3].

The liquefaction phenomenon was recorded at Pohang for the first time in South Korean history. Liquefaction effects, such as sand boil and cracks, were observed; these caused damage to schools and residential buildings. This scenario indicates the necessity for increased geological and geotechnical attention toward liquefaction susceptibility because of seismic hazards [4]. The factors that induce liquefaction include the geotechnical properties of the ground, topography, soil type, age, sedimentation process, the granularity of the soil, density, distribution, burial depth of soil, slope, depth of water table, and geological history [5]. The ground failures caused by liquefaction are the reasons for most of the damage during and after earthquakes. Volume losses in the liquefied soil during and after earthquakes result in ground surface settlement. Settlement causes instability and damage to the building structures if the surface settlement is not uniform throughout the building structures. The liquefaction induces vertical and horizontal displacements in building structures with shallow foundations. The differences in vertical and horizontal displacements allow the building structures to be sustained on shallow foundations. Structures with deep foundations are typically associated with flow sliding and lateral spreading [6].

The Pohang earthquake had destructive consequences and caused lateral liquefaction spreading. The relationship between the soil liquefaction and loading during the earthquake was studied at laboratories using (among other equipment) the shake table, cyclic triaxial, centrifugal, and resonant column tests [7]. Research on settlements after the liquefaction phenomenon will continue to be conducted to prevent damage vulnerabilities. The liquefaction-induced settlements at Pohang were predicted using numerous analytical and numerical methods [8]. Assessing the settlements of liquefaction is associated with many challenges, such as the resedimentation of liquefied soil, zero volumetric changes, and ground loss because of sand boils, ratcheting, and bearing capacity failure. Predicting the potential of liquefaction in the soil is the most critical part of assessing the liquefaction-related damage. The susceptibility of soil toward liquefaction was determined using simplified liquefaction triggering procedures on readily available free-field data. The standard penetration test (SPT) and cone penetration test (CPT) are the commonly implemented methods that use the shear velocity of the soil and other testing tools. The selection of field-testing tools depends on their availability and costs. Not all devices are suitable for all scenarios because each device has its advantages and drawbacks. Owing to the availability of SPT and CPT instruments, methods based on these are implemented extensively [9].

Several numerical analyses have been conducted by coupling soil and building bases with or without superstructures. A centrifugal study of an earthquake was conducted by loading the soil models and inducing liquefaction to explore the mechanisms of liquefaction, parameter acquisition, and analysis of the procedure used for liquefaction problems [10]. A mathematical theory using the LaGrange motion equation and the Hamiltonian principle was proposed to estimate the soil displacement due to earthquakes. The model predicted scalar soil displacement with time, but it performed well only with a limited number of inputs [11]. Three-dimensional numerical simulations on finite element models are found to provide an accurate estimation of liquefaction-induced settlements and deformation. Centrifugal experiments were conducted with varied soil profile configurations and single degrees of structures with different earthquake motions. The results showed that volumetric strains are due to partial draining during an earthquake, and deviatoric strains are due to bearing capacity failure and soil structure interaction-induced building ratcheting. A numerical analysis was conducted on soil nonlinearity caused by liquefaction and its interaction between the structural foundation using a finite element model, preloading simulation, boundary condition, and structural model. The study proved that the soil structural characteristics are modified by soil foundation mitigation; preloading reduces structural settlement, and the mitigation method reduces the liquefaction probability. Experimentations with nine node quadrilateral elements, Lysmer–Kuhlemeyer dashpot, and columns of soil with boundary conditions were conducted to estimate the rigidity of the elastic medium. The analysis showed that there is increased vulnerability of lateral spreading in partially submerged slope grounds. Computation modeling was implemented to study the liquefaction-induced effects on free-field response and stone column mitigation techniques. The study confirmed that stone column remediation effectively reduces lateral deformation. Liquefaction estimation in free-field settlements was conducted using two-dimensional fully coupled finite element analysis and observed to perform better than semi-empirical methods. The Plaxis 2D and PM4Sand finite code elements were implemented to calculate liquefaction, and the results were compared with semi-empirical methods. The results showed that the PM4Sand-Model had lower settlement values than the semi-empirical methods [12]. A probabilistic numerical framework using a fully coupled formulation was implemented to study the soil characteristics based on volumetric soil strains and pore water pressure. The results obtained from the numerical analysis were used for predicting post-earthquake settlement using the Bayesian linear regression model [13]. Most of the existing procedures used to assess the liquefaction-induced settlements were conducted by considering the free fields without buildings. These procedures neglect the effects of stress and load on the soil of building foundations caused by soil liquefaction on the building. Most of the analytical and numerical studies have failed to investigate the soil structures and ground motion properties [14].

The liquefaction of soil because of seismic activity is more complex and is dependent on numerous factors. Therefore, it is extremely difficult to predict soil liquefaction considering the entire factor set. Methods such as the CPT and SPT have been employed to identify the relevant factors of soil liquefaction. A review on 203 articles about soil liquefaction and its 22 factors was conducted. Based on an indicator, a single literature impact factor, which is evaluated using the impact factor of the article and is the most studied seismic factor, the significant factors were selected. Among 22 factors studied, 12 factors were considered to be significant; these included the epicenter, magnitude, particle content and size, relative density, grain composition, duration, degree of consolidation, thickness and sand layer depth of soil layer, groundwater table, and drainage condition. The major factors and their relationships were determined using interpretive structural modeling (ISM), where the epicenter distance, magnitude, drainage condition, and duration influenced the liquefaction, and played critical roles. The results provide strategies for the selection of the features and the construction of a model to predict soil liquefaction [15]. Owing to the high uncertainty of soil characteristics and earthquake sites, choosing an empirical formula for regression analysis becomes challenging.

Recently, studies based on machine learning (ML) models have attempted to predict soil liquefaction [16]. ML methods such as support vector machines (SVMs), relevant vector machines, and artificial neural networks (ANNs) have been applied to predict earthquake liquefaction. Seismic liquefaction potential based on the dataset of earthquake cases in Turkey and Taiwan was evaluated using an ANN. Evaluation of the soil liquefaction potential was conducted using a fuzzy neural network, which was used to test more than 11 earthquake datasets acquired between 1964 and 1999 [17]. Complex correlations between liquefaction factors and the seismic properties were established using genetic expression programming [18].

A neural network with CPT data-based empirical equation was developed. Earthquake data collected by various laboratory studies were used to predict the liquefaction cyclic resistance ratio (CRR) using backpropagation (BP) neural networks. An RVM model was developed on SPT data from the Chi–Chi earthquake for determining the potential of liquefaction. The potential of an SVM with CPT- and SPT-based data was investigated and compared with the ANN model. A dataset with 466 parameters of field liquefaction performance and CPT measurements was used for training and testing the SVM model, which managed to produce an accuracy of 98.71% [19]. The SVM-based model proved to be effective and flexible compared with the ANN as the SVM requires only two parameters, whereas the ANN requires numerous parameters.

Although ML algorithms are more effective than statistical methods, they suffer from overfitting, low convergence, and poor generalization problems [20,21]. Kernel-based methods, such as the SVM and RVM, are associated with numerous challenges, such as kernel selection, kernel parameters, and interpretation of the model [21]. Bayesian network models were used to impute the missing data of incomplete datasets. Tree-based models were used to address the overfitting problems in the prediction of the liquefaction potential of soil. Although the ML models produced an accurate prediction on a specific dataset, the performance of the models was not high for other liquefaction datasets [22].

These drawbacks can be addressed by building hybrid models through the stacking of good prediction models [23]. Although the SVM can minimize structural complexity, it cannot reduce the errors in the prediction of liquefaction. Particle swarm optimization (PSO) was used in conjunction with an SVM to improve its prediction efficiency by tuning its hyperparameters [24]. The ANN was used with BP to increase the competency of the prediction models. Furthermore, optimization algorithms such as genetic and particle swarm algorithms were implemented in BP neural network models to elevate performance. Hybridized models, such as BP-GA and BP-PSO, were developed, and the model efficiencies were compared. BP-PSO was observed to be a reliable prediction approach subject to earthquake uncertainties [25].

From the literature, it is evident that an aggregation of performing models would upgrade their prediction efficiency [26]. Ensemble models produce a higher accuracy than an individual model and can be applied to linear and nonlinear datasets. The ensemble method improves the robustness of the prediction model [27]. This proposed work builds an efficient aggregate model by stacking multilayer perceptron (MLP), support vector regression (SVR), and linear regression (LR) to predict the liquefaction-induced settlement at Pohang using available SPT data obtained from the Korea geotechnical information database system.

The proposed model aims to: (a) build an intelligent predictive model for estimating earthquake-induced liquefaction settlement to overcome the drawbacks of traditional methods in measuring earthquake-induced soil liquefaction; (b) develop a stacked generalization model to enhance the accuracy of earthquake-induced soil liquefaction; (c) impute the new data points in the dataset to enhance the volume and diversity of available features using tabular generative adversarial networks (TGANs).

The organization of this paper is as follows: Section 2 discusses the dataset, data augmentation, and analysis of the actual and augmented dataset. Section 3 describes the general stacking methodology, architecture of the SVR, MLPR, and the proposed SVG model. Section 4 discusses the evaluation metrics and the performance comparison between the SVR, MLPR, and SGM. Conclusions and scope for enhancement are presented in Section 5.

## 2. Data Modeling

Liquefaction is inferred to be one-dimensional along with the vertical soil layers, where the earthquake produces cyclic shear and compressive forces. Hence, a pore pressure is created along with the inner soil layer, reducing its stiffness and strength. Reconsolidation occurs on liquefaction as the pore pressure is exerted on the water from the soil, which reduces its volume and induces the vertical settlement of the ground. The approximation of the occurrence of liquefaction is important for estimating the damage associated with it. The parameters that measure the potential of soil liquefaction are shear velocity (Vs), the cyclic stress ratio (CSR), and CRR. The triggering of liquefaction is measured using CPT-based triggering and simplified liquefaction triggering methods. Simplified liquefaction triggering is considered the best practical tool for measuring the susceptibility of liquefaction. The SPT is a commonly adapted procedure that quantifies geotechnical engineering properties, such as relative density, granularity, and bearing capacity of the subsurface soil. In this study, the proposed stacked generalization method was modeled using the SPT dataset. A data augmentation technique was applied to the dataset to increase its volume and diversity.

### 2.1. Dataset Description

**Figure 1:**
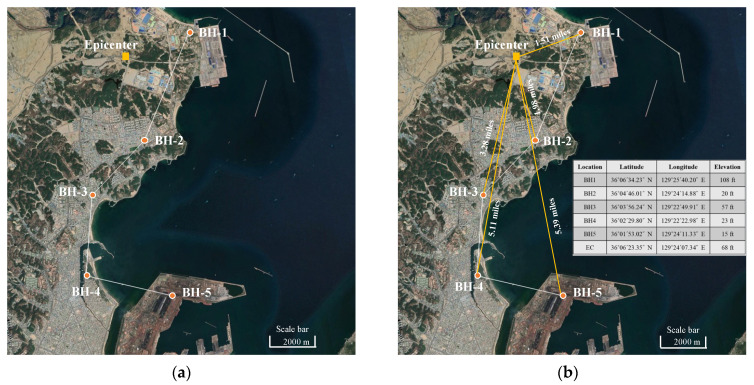
Geographical locations of epicenter of Pohang earthquake considered in this study. (**a**) Locations of epicenter and borehole; (**b**) geographical details of locations.

The dataset used for the study was obtained from the Korean Geotechnical Information DB system [28] and a fully coupled stress model [29]. The one-dimensional column analysis was conducted on an effective stress model, which evaluated the shear-induced deformation from earthquake- and SPT-based data [30]. Figure 1a shows the five locations of the borehole (BH-1, BH-2, BH-3, BH-4, and BH-5) around the epicenter (EC), and the geological details of the Pohang earthquake, based on which the SPT database was created. The distance between the EC and the different borehole locations from BH-1 to BH-5 was measured and found to be 1.51 miles, 1.98 miles, 3.28 miles, 5.11 miles, and 5.39 miles, respectively. The geographical attributes of the boreholes and EC are represented in Figure 1b.

Five influencing parameters, including the soil layer depth in m, unit weight (kN/m^3^), CSR, induced liquefaction settlement in mm, and corrected standard penetration blow numbers (N1(60)), were collected from 20 records on each borehole, thus generating approximately 100 records. A safe threshold value of N1 was identified as 60; the N1 value was corrected to a normalized N1(60). The dataset contained approximately 20 data points along with the corresponding settlement values for each borehole; this generated 100 data points. Table 1 details the statistical summary of the dataset.

### 2.2. Data Augmentation

Data augmentation is the process of synthesizing new data from existing data. A model based on the minimum number of data points can lead to over/underfitting. A model with sufficient and rich data performs efficiently with better predictive accuracy. Supplying a good quantity of data may reduce the training errors and improve the overall performance of the model [31]. It is important that the data are in an algorithm-recognizable tabular format as the performance of the stacked generalization approach depends on the quantity of the supplied data. As the SPT dataset’s size considered for building the proposed liquefaction prediction model is small, it requires data augmentation to create an efficient model. As the available number of SPT data points is minimum, data augmentation was performed to synthesize some additional data to enhance the performance of the proposed model.

Tabular generative adversarial networks (TGANs) utilize the power of deep neural connections to produce high-quality and factitious tables by generating discrete and continuous values [32]. TGANs concentrate on the generation of tabular data to ensure that the training data distribution is close to the test data distribution. Sampling training data train the proposed model using adversarial training. The model’s performance is compared using the initial training dataset and with TGAN-generated data to validate the data augmentation methodology.

**Figure 2:**
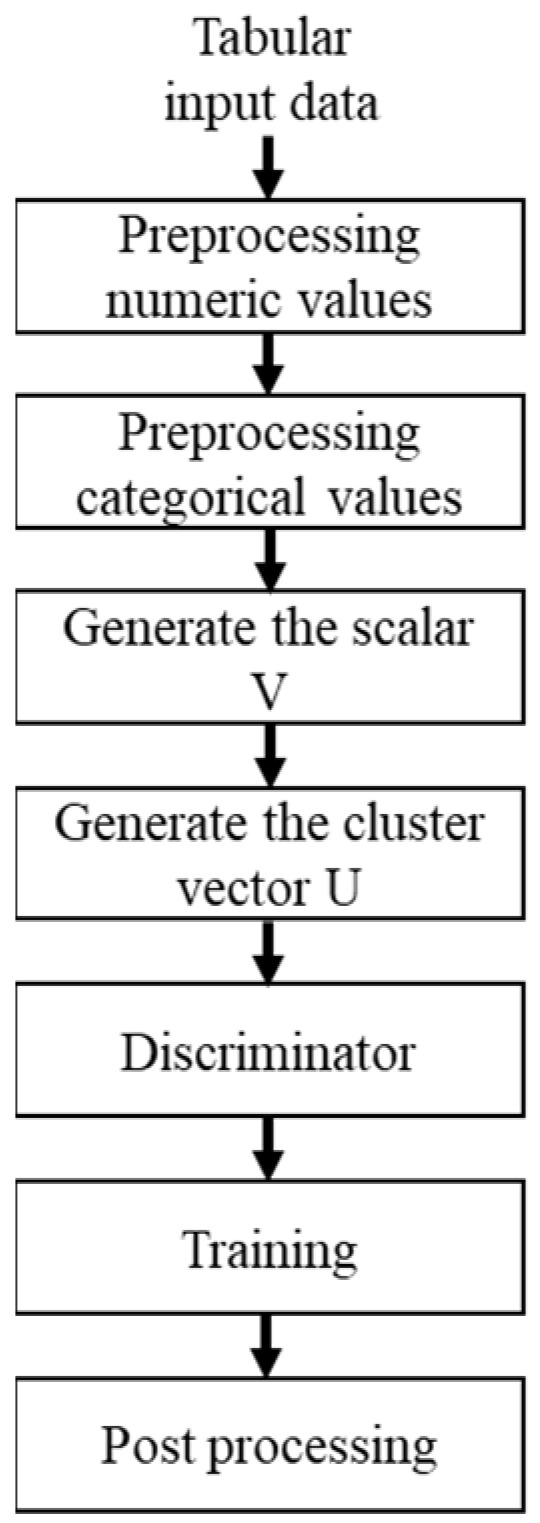
Tabular generative adversarial network (TGAN) working procedure.

The actual SPT dataset (T) used in the proposed study consisted of three discrete features (n_d) and two continuous features (n_c). R represents each row in the table in the form of a vector in an n-dimensional space. P denotes the unknown joint distribution between the features. The main objective of a TGAN is to train an imputation model (IM). The IM should generate a high-quantity factitious table (F) with a data distribution similar to T. Building a stacking model based on F will achieve the maximum performance compared with those of the existing models in the literature [33]. Figure 2 depicts the working procedure of a TGAN.

**Figure 3:**
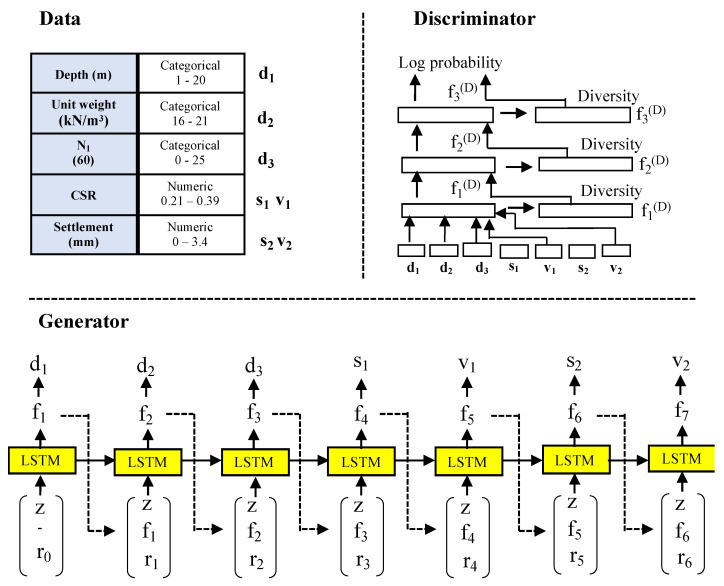
TGAN architecture.

The Gaussian mixture model (GMM) was applied to the TGAN for preprocessing the data points in R. The GMM generates suitable data points with multimodal data for each feature in R. The GMM is used to normalize R to generate S. The GMM computes the probability of R during each Gaussian iteration as a vector V. After preprocessing, the TGAN converts the table T with discrete and continuous data points to S, V, and D vectors. These vectors are the generator’s output and are fed as the input to the discriminator in the TGAN. Figure 3 shows the detailed architecture and internal working of the TGAN.

The generator produces a numerical variable in two steps and categorical data in a single step. Initially, a scalar value S is generated; eventually, upon the application of the function tanh on S, a cluster vector V is produced [34]. The categorical variables are generated based on the probability distribution of all possible labels with the softmax function. Long short-term memory (LSTM) is used as a generator to synthesize the dataset. A random variable z, previous hidden vector f_i_, and weighted context vector r_i_ are used as the input to LSTM in each step i.

The discriminator in the TGAN is configured by integrating the MLP with the leaky rectified linear activation unit (ReLU), and BatchNorm is used. The discriminator concatenates the vectors S, V, and D that are produced by the LSTM generator. The Adam optimizer is applied to optimize the KL divergence of discrete and continuous variables. The generator produces synthesized data for each data point at each step. The discriminator concatenates the actual and synthesized data. MLP with leaky ReLU is used to differentiate real and synthesized data. The generator is trained to produce better synthesized data, whereas the discriminator is trained to differentiate between actual and synthesized data. The non-Gaussian and multimodal distribution of data are handled by applying mode-specific normalization distribution over the TGAN. After training, the model is executed to synthesize data. The generator produces the synthesized data, which cannot be differentiated by the discriminator [35].

### 2.3. Analysis of Data Distribution

**Figure 4:**
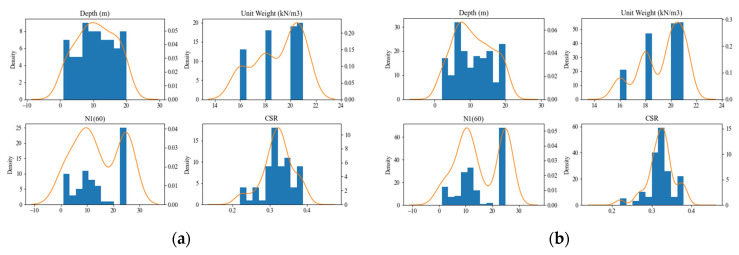
Feature distributions of actual and augmented data. (**a**) Actual data; (**b**) augmented data.

**Figure 5:**
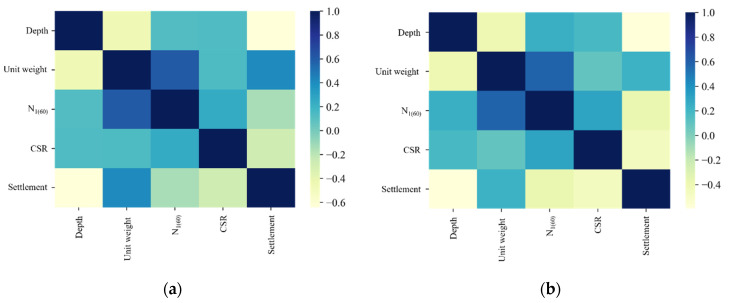
Heat map visualizations of data features. (**a**) Actual data; (**b**) augmented data.

The actual benchmark dataset on liquefaction attributed to earthquakes collected from the Korea Geotechnical Information holds 100 data points distributed over five features. A data augmentation technique using the TGAN approach is proposed to enhance the performance of the ML models to predict the liquefaction-induced settlement. The TGAN managed to augment 177 data points, and the distribution of the data points is listed in Table 2. After data augmentation, the magnitude of the dataset increased 1.7-fold with reasonable data distribution. Comparison of the mean and standard deviation (SD) values in Table 1 and Table 2 show that the data augmented by the TGAN were in line with the actual data. The feature distribution and heat map visualization of dataset features are visualized in Figure 4 and Figure 5, respectively. The heatmap visualization was applied in the proposed work to visualize the correlation between the features of multivariant data. Figure 5 is presented to show the comparison between the heatmap of original data and the heatmap of augmented data. As shown in Figure 5, both the heatmaps look very similar, thereby proving that the correlation between the feature set remained unchanged and untainted by the data augmentation process. Finally, 277 data points obtained after data augmentation were used to build the SGM for liquefaction settlement prediction.

## 3. Materials and Methods

### 3.1. Stacking Generalization

Stacking generalization is an ensemble methodology proposed to reduce the error rate with more generalizations over the ML models. Stacking concentrates on combining the results of two or more models on the same dataset. Here, each ML model is defined as a base model and assembled over the meta-model for generalization. Base models concentrate on defining the mathematical function of the training data and observe the estimation. The stacking platform integrates the estimates of base models, and the integrated result is used as an input for the meta-model. Subsequently, the meta-model combines the estimations of the base models. The meta-model defines an integration process for deriving the generalized architecture [36]. In stacking, the base models are often complex and diverse. It is an excellent approach to choosing models that are very different in their core intuitions and capable of observing the diversified estimates. The meta-model is simple and performs straightforward predictions for the actual problem definition.

The stacking ensemble predictor was formulated in this study by observing the predictions using two base models. The observations were integrated by using a meta-model. We used SVR and multilevel perceptron regression (MLPR) as base models to predict the liquefaction settlement. LR was chosen as a meta-model for integrating the observations of the base models. Using a simple linear model as the meta-model often gives a blended stacking of base models. The strengths and weaknesses of the base model and the me-ta-model applied in this proposed approach are summarized in Table 3. The following sections (Section 3.2 and Section 3.3) describe the basic operations of the base model architectures involved in this study. Stacking of the base models is discussed in Section 3.4.

### 3.2. SVR Base Model Architecture

SVR is a supervised learning algorithm applied for prediction tasks. The basic working principle behind SVR is based on the identification of the optimum fit line for the data distributions. The optimum fit line in SVR is recognized as the hyperplane that has the highest number of feature points. The commonly used regression models reduce the sum-of-squared errors. In contrast, SVR tries to optimize the hyperparameters to fix the optimal hyperplane for the given data distribution. The optimal hyperplane in SVR has a maximum margin between the boundary line and the distributed feature points.

In the SVR problem formulation, the total error is less than or equal to a particular marginal value and is specified as the maximum error, and is denoted as ϵ. The complete error term (ϵ) is included as a constraint while determining the objective function. The objective function in SVR always tries to fit the maximum possible features on the best possible hyperplane to fit the maximum number of data points on the hyperplane. When the error value is more significant than ϵ, there is a possibility that the data points will fall outside the margin. The concept of a slack variable is used to address this limitation in SVR. For any feature that falls outside ϵ, the deviation that occurs from the margin is denoted as ξ.

**Figure 6:**
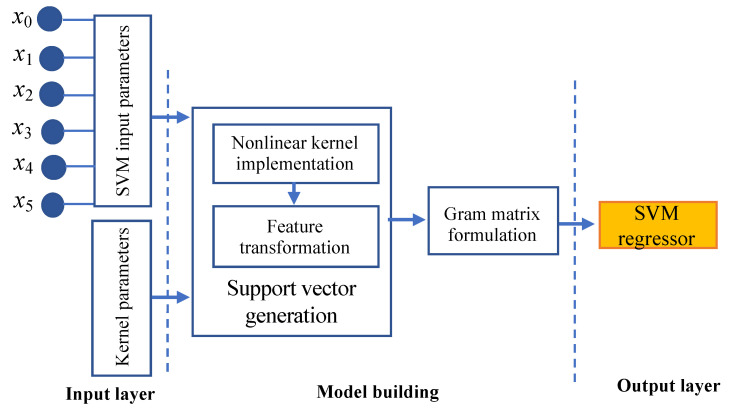
Architecture of support vector regression (SVR).

The internal working architecture (Figure 6) of SVR comprises three layers: input layer, model building, and output layer. The input layer uses the entire feature set available in the dataset as the SVM input parameter set. For a nonlinear data distribution, the kernel parameters are used as the input for model construction. The model construction process concentrates on the implementation of the nonlinear kernel function followed by feature transformation for generating the support vectors. The gram matrix is formulated from the generated support vectors as part of the model construction process. The SVM regressor will estimate the predictor variable in the output layer.

The training liquefication dataset in this proposed study included the different feature variable $X_{n}$ and observed settlement variable s_n_. The predictor variable $X_{n}$ was represented as $X_{n}$ = {x_1n_, x_2n_, x_3n_, x_4n_, x_5n_}. The objective of the SVR was to generate a function F(X) that deviated from the settlement variable s_n_ by a value not exceeding the maximum error (ϵ) for each data point in X. The linear function and the criterion for formulating the objective function are listed in Equations (1) and (2), respectively.

(1) $$F\left(s_{n}\right)={\;X}_{n}\beta +c,$$

(2) $$\forall n\;:\left|s_{n}-\left(X_{n}\beta +c\right)\right|\leq \;\epsilon +\xi ,$$

where n represents the total number of observations in the dataset, $X_{n}$ is the predictor variable, and s_n_ is the settlement variable. The symbols β, ξ, and c represent the slope, slack variable, and intercept of the hyperplane, respectively. As the proposed problem is defined by the nonlinear data distribution, the kernel function can be applied to transform the nonlinear data points into higher dimensions. The kernel function applied in the nonlinear SVR is represented by Equation (3).

(3) $$G(X_{n})=\text{<}\varphi (X_{n})>,$$

where φ($X_{n}$) is the conversion function that maps X into a higher dimensional space. The gram matrix and polynomial kernel function applied in SVR for the higher dimensional space transformation are represented by Equations (4) and (5), respectively.

(4) $$G_{i,j}{=G\;(X}_{i}{,\;X}_{j}\;),$$

(5) $$G(X_{i},\;X_{j})={\left(1+X_{i}{,\;X}_{j}\right)}^{q}\;\forall \;q\;in\;\{2,\ldots n\},$$

The gram matrix is formulated as a matrix with elements in n rows and n columns corresponding to g_i,j_. Every aspect of the gram matrix, g_i_,_j_ is equivalent to the inner product of the features altered by *φ*. The gram matrix supports SVR to fix an optimal function ${F(s}_{n})$ in the altered predictor space. The procedure anticipates the new values depending only on the support vectors. Hence, the Lagrangian function used for optimization is defined in Equation (6), by presenting the nonnegative multipliers α_n_ and α^*^_n_ for every feature in X.

(6) $$F\left(s_{n}\right)=\sum_{n=1}^{N}\;\left(\alpha_{n}-\alpha_{n}^{*}\right)\;G\left(X_{i}{,\;X}_{j}\right)+b,$$

where α_n_ and α^*^_n_ are nonnegative multipliers. The predicted value from the SVR base model is subsequently used as an input to the meta-model in the stacking architecture to enhance the prediction accuracy.

### 3.3. MLP Regressor

MLP is an ANN-based algorithm that learns the transformation function F(X). The function F(X) can represent an m-dimensional space on the training dataset as F(X): R^i^ → R^o^, where the total number of input features is denoted as i and the total number of output features is denoted as o. The MLP regressor (MLPR) implements the MLP algorithm for the prediction task trained by using the backpropagation method. The internal architecture of the MLPR is formed by integrating the input layer with hidden layers and the output layer.

The MLPR was trained using the backpropagation approach for regression tasks with no activation function in the output layer. The identity fction was used as an activation function in the output layer. Given that the expected predictor value was continuous, the squared error was applied as the loss function, and the parameter alpha (α) was applied as a regularization term. This helps overcome the overfitting issues by penalizing weights with large magnitudes. The regularization term α resists overfitting by restricting the weight values. Increasing α may fix the variance to a high value by promoting lesser weights, thus resulting in a feature space with fewer curvatures.

**Figure 7:**
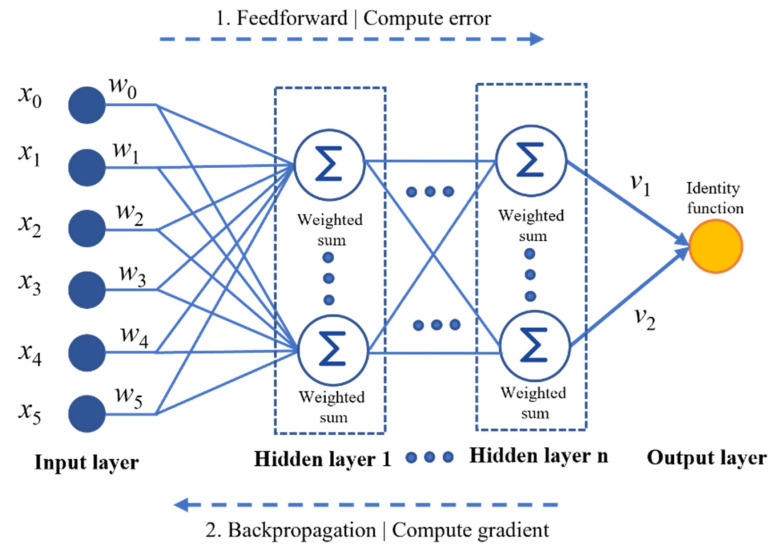
Architecture of multilayer preceptor regressor (MLPR).

The MLPR architecture configured for the stacked architecture is represented in Figure 7. A set of neurons in the input layer focuses on representing the X_n_ input features in the dataset. Assume the existence of a set of features in an n-dimensional space $X_{n}$ and a target feature $y_{n}$, where $X_{n}$ ε R^n^ and $y_{n}$ ε R^m^. The individual feature vectors in an n-dimensional space can be defined as (X_1_, y_1_), (X_2_, y_2_), …, (X_n_, y_n_). The feature vector X for the SPT dataset is represented as X_n_ = {x_n1_, x_n2_, …, x_nn_}. The input features are transformed in the hidden layer by performing the linear summation of the input features with the weight values. Each neuron performs the weighted linear summation (WLS) followed by a nonlinear activation function, as represented by Equations (7) and (8).

(7) $$\mathrm{WLS}\;\left(W_{n}{,\;X}_{n}\right){=w}_{1}x_{1\;}{+w}_{2}x_{2\;}{+\ldots \;w}_{n}x_{n\;},$$

(8) $$g\left(\cdot \right)\;:{\;R}^{i}\to R^{o}.$$

For regression problems, the output from the multiple linear regression (MLR) is F(x), and the output is the identity activation function (Equation (9)).

(9) $$F\left(x\right){=W}_{2}\;g\;\left(W_{1}^{T}{x+p}_{1}\right){+p}_{2},$$

where $W_{1}\in {\;R}_{m}$ and $W_{2}{,\;p}_{1,\;}p_{2}\;\in \;R$ are the proposed model parameters. The weight values of the interconnected neurons in the input and the hidden layers are represented as W_1_ and W_2_, respectively. The parameter b_1_ is included as the bias value to the hidden neurons, and b_2_ is added as the bias value to the output neuron. The identity function at the output layer is represented as g. For the prediction tasks, the output function should remain as F(x). Hence, the identity function is integrated as an activation function at the output layer. In this study, the loss function is applied at the output layer to determine the variance between the actual and the expected settlement value from the MLPR. The essential gradients used to update the weights of internal nodes are derived using the loss function. The square error loss function mentioned in Equation (10) is applied to derive the gradients.

(10) $$\mathrm{Loss}\;\left(\;S_{P},\;S_{A},\;W\right)=\frac{1}{2\;}\;||\;S_{P}\;-\;S_{A}\;||\begin{array}{c} 2\\ 2 \end{array}+\frac{\alpha }{2\;}\;||w||\begin{array}{c} 2\\ 2 \end{array},$$

where $S_{P}$ represents the predicted liquefaction settlement, and $S_{A}$ represents the actual liquefaction settlement. W represents the weight value, and $\frac{\alpha }{2\;}\left||w|\right|\begin{array}{c} 2\\ 2 \end{array}$ is the L2 regularization term. The MLPR fine-tuned the initial random weights to minimize the loss function by repeatedly updating the weight values. Once the loss function was computed, the backpropagation was initiated from the output layer to the hidden layers. Every weight value was updated during backpropagation to decrease the overall loss value. The gradient descent approach was applied to measure the gradient loss (∇Loss) with respect to the computed weight value. The weight value was adjusted based on the gradient loss as indicated by Equation (11).

(11) $$W^{i+1}{=W}^{i}-\;\in \;\nabla \;\mathrm{Loss}\begin{array}{c} i\\ W \end{array},$$

where the iteration is represented as i, and ∈ represents a learning rate greater than 0. $\nabla {\;\mathrm{Loss}}_{w}$ is the gradient loss with respect to the computed weights. During the MLPR execution, the gradient loss is computed iteratively, and the algorithm stops when it reaches a maximum iteration number defined a priori.

### 3.4. Stacking the Base Models

**Figure 8:**
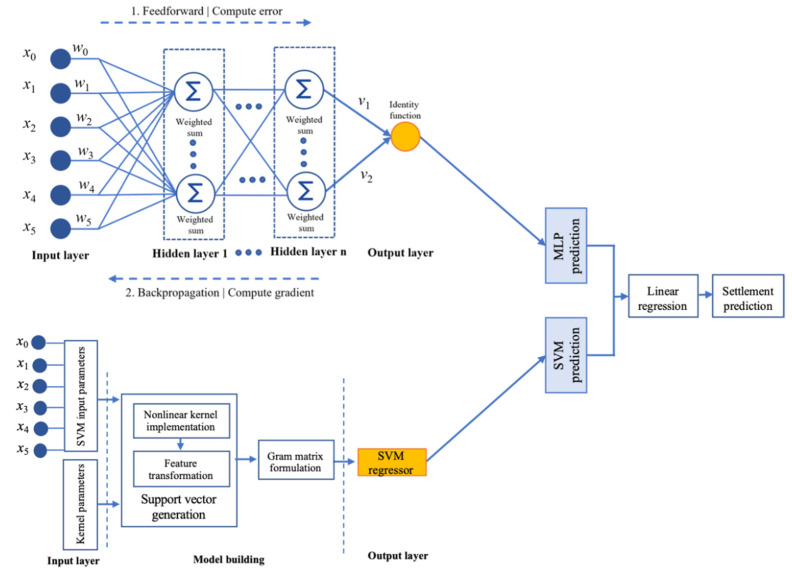
Architecture of stacked generalization model (SGM).

Stacked generalization concentrates on the integration of the predictions from two or more base models to enhance the overall performance of the ML models [37]. Stacked generalization harnesses the capabilities of the well-performing ML models to ensure that estimates will have a better combined accuracy than the individual model [38]. In this study, stacking was performed based on the predictions on MLP and SVR. MLP depended on the hidden layers and the identify function to extract the essential features from the input data. The fully connected network integrated the extracted features, and the output layer made the prediction. In this liquefication dataset, MLP took advantage of the hidden nodes, weighted sum, and gradient computation, but also explored the relationship between the input features in the higher dimensional space efficiently [39]. The role of the kernel function for mapping the features into the higher dimensional space helps generate the support vector formation in SVR. The kernel function a the gram matrix formation in SVR helps enhance the accuracy of liquefaction prediction in SVR. To further improve the effectiveness of the forecast for the liquefaction dataset, the stacking of the MLP and SVM on the MLR model was proposed. The fully connected stacking architecture of MLP and SVR over MLR was defined as a stacked generalization model (SGM), as shown in Figure 8.

MLR was applied as a generalizer to combine the predictions from the MLP and SVR. The LR assigned one scale factor to each predicted value, commonly referred to as the coefficient β. MLR fitted a multiparameter model with multiple β coefficients, where β is distributed as {β_1_, β_2_}. The coefficient concentrates on minimizing the sum-of-squared residuals between the actual and observed settlement values based on linear approximation. The general expression for combining the prediction is given by Equation (12).

Y = β_0_ + β_1_ X_1_ + β_2_ X_2_ + ε, (12)

where Y represents the predictor variable of stacked architecture, X_1_ represents the predictor variable of MLP, X_2_ represents the predictor variable of SVR, β_0_ represents the intercept, and β_1_ and β_2_ represent the regression coefficients.

The multilayer preceptor regressor (MLPR) configured for the proposed model has a single hidden neural layer containing eight neurons for straightforward computation of predictions. Each neuron possesses weight, and a bias is randomly initialized using the Xavier initialization method. The method produces random numbers with uniform probability distribution between the upper and lower bounds of the data points. During training, the batch size is configured as eight to optimize the loss function. Algorithm 1 explains the process of SGM liquefication prediction.
**Algorithm 1:Stacked generalization model (SGM) for liquefication prediction**1. Let X be the input features in liquefication dataset D and y be the label for X in D X_n_ = {x_n1_, x_n2_, …x_nn_}, where $X_{n}$ ε R^n^ and $y_{n}$. ε R^m^ D has n_d discrete features and n_c continuous features; 2. Perform data augmentation on the available data features using a tabular generative adversarial network (TGAN) a. Apply the GMM to the TGAN for preprocessing b. Configure generator (G) and discriminator (D) for the TGAN c. for r = 1 to n data points TGAN imputation repeated for r times Generator (G): Generate the scalar S, cluster vector V, and D vector by applying the TGAN Discriminator (D): Integrate MLP with LeakyReLU and Batch Norm Synthesize (S): Generate input values end for 3. Initialize SGM Base model: 02: SVR and MLPR Meta-model: 01: MLR 4. Build SVR base model a. Define objective function: $F\left(s_{n}\right)={\;X}_{n}\beta +c$, $\forall n\;:\left|s_{n}-\left(X_{n}\beta +c\right)\right|\leq \;\epsilon +\xi$ b. Apply kernel function: G($X_{n}$) = <φ($X_{n}$)> c. Transform gram matrix: $g_{i,j}{=G\;(X}_{i}{,\;X}_{j}$) d. Generate polynomial kernel function: G(X_i_*,* X_j_) = (1 + $X_{I}{,\;X}_{j}$*)^q^*
∀ q in  {2,…n} e. Finalize SVR predictive function: $F\left(s_{n}\right)=\sum_{n=1}^{N}\;\left(\alpha_{n}-\alpha_{n}^{*}\right)\;G\left(X_{i}{,\;X}_{j}\right)+b$ 5. Build MLPR base model a. Define linear weighted summation: $\mathrm{WLS}\;\left(W_{n}{,\;X}_{n}\right){=w}_{1}x_{1\;}{+w}_{2}x_{2\;}{+\ldots \;w}_{n}x_{n\;}$ b. Apply nonlinear activation function: $g\left(\cdot \right)\;:\;R^{i}\to R^{o}$, where *i: input features, o: output features* c. Activate identity function: $F\left(x\right){=W}_{2}\;g\;\left(W_{1}^{T}{x+b}_{1}\right){+b}_{2}$ d. Apply loss function: $\mathrm{Loss}\;\left(\;S_{P},\;S_{A},\;W\right)=\frac{1}{2\;}\;||\;S_{P}-\;S_{A}\;||\begin{array}{c} 2\\ 2 \end{array}+\frac{\alpha }{2\;}\;||w||\begin{array}{c} 2\\ 2 \end{array}$ e. Deploy Gradient decent function: $W^{i+1}{=W}^{i}-\;\in \;\nabla \;\mathrm{Loss}\begin{array}{c} i\\ W \end{array}$ 6. Stacking meta-model a. Integrate predictions of SVR and MLP b. Combine prediction: Y = β_0_ + β_1_ X_1_ + β_2_ X_2_ + ε

## 4. Results and Discussion

The test results from SPT were used to evaluate the liquefaction-induced settlement. The data augmentation technique, which used a TGAN, was applied over the actual dataset to increase the dataset volume. A total of 277 SPT data instances, each with five attributes, were considered in this study. Among the different features in the dataset, the settlement remained a class-labeled feature for the construction of the liquefaction-induced settlement prediction model. For building the SGM model, a 70–30 train test scheme was applied to improve the model performance. Approximately 193 instances of SPT data were used for training, and 84 cases were used as validation data. The different performance metrics considered in this study are discussed in the following section.

### 4.1. Performance Evaluation Metrics

The execution of the SGM was evaluated by using a different system of measurement, namely the R^2^ score, mean-square error (MSE), standard deviation (SD), covariance (COV), and the root-mean-square error (RMSE). The R^2^ score indicates the dependency between the independent and the dependent features in a dataset. The R^2^ score represents the best line of fit for the actual settlement and the predicted settlement values. The greater the value of the R^2^ score, the better the line of fit between the actual and predicted values. In settlement prediction, the SD plays a vital role in measuring the settlement data’s dispersion relative to its mean value. SD is measured as the square root of the variance. The R^2^ score can be calculated using Equation (13). The procedure for measuring the SD for the actual and predicted data points is expressed by Equations (14) and (15), respectively.

(13) $$R^{2}=1-\frac{\sum_{1}^{n}{{(s}_{i}-{\hat{s}}_{i})}^{2}}{\sum_{1}^{n}{{(s}_{i}-\bar{s})}^{2}},$$

(14) $$\mathrm{SD}\;(\mathrm{Actual})=\sqrt{\frac{\sum_{i=1}^{n}{{(s}_{i}-\bar{s})}^{2}}{n-1}},$$

(15) $$\mathrm{SD}\;(\mathrm{Predicted})=\sqrt{\frac{\sum_{i=1}^{n}{\left({\hat{s}}_{i}-\bar{s}\right)}^{2}}{n-1}},$$

where $s_{i}$ represents the actual settlement value of the *i*th instance, ${\hat{s}}_{i}$ is the estimated settlement value of the *i*th instance, and $\bar{s}$ represents the mean of all actual settlement values taken into consideration.

Covariance represents the total variation in the observations between the original settlement and the estimated settlement values. A constructive association between the settlements indicates that the actual and estimated values are close to each other. A destructive association between the settlements implies that the actual and estimated values find it difficult to deliver the best fit. Equation (16) represents the COV assessment between the actual and predicted settlement values.

The mean square measures the closeness of the estimated best-fitted line to the actual data points. The vertical distance between the data point and the estimated best fitted line is referred to as the residual error. The residual error is evaluated and squared for all the data points. The estimated error values’ average is calculated as the MSE. The smaller the MSE value, the closer the model’s fit to the data. In addition, RMSE represents the dispersion of the residual functions. RMSE is represented as the square root of MSE. MSE and RMSE are estimated based on Equations (17) and (18), respectively.

(16) $$\mathrm{COV}=\frac{\sum_{i=1}^{n}{(s}_{i}-\bar{s})\left({\hat{s}}_{i}-\bar{s}\right)}{n-1},$$

(17) $$\mathrm{MSE}=\frac{1}{n}\sum_{i=1}^{n}{{(s}_{i}-{\hat{s}}_{i})}^{2},$$

(18) $$\mathrm{RMSE}=\sqrt{\frac{1}{n}\sum_{i=1}^{n}{{(s}_{i}-{\hat{s}}_{i})}^{2}},$$

where $s_{i}$ represents the actual settlement value of the *i*th instance, ${\hat{s}}_{i}$ is the estimated settlement value of the *i*th instance, and ${\hat{s}}_{i}$ represents the average of all actual settlement features taken into consideration.

### 4.2. Performance Comparison

In this study, an SGM was developed by stacking the MLPR and SVR over the regression model. A comparative study was conducted with the base models, namely MLPR and SVR, to validate the performance of the SGM. Table 4 summarizes the performances of different models used in this study based on performance evaluation metrics.

**Figure 9:**
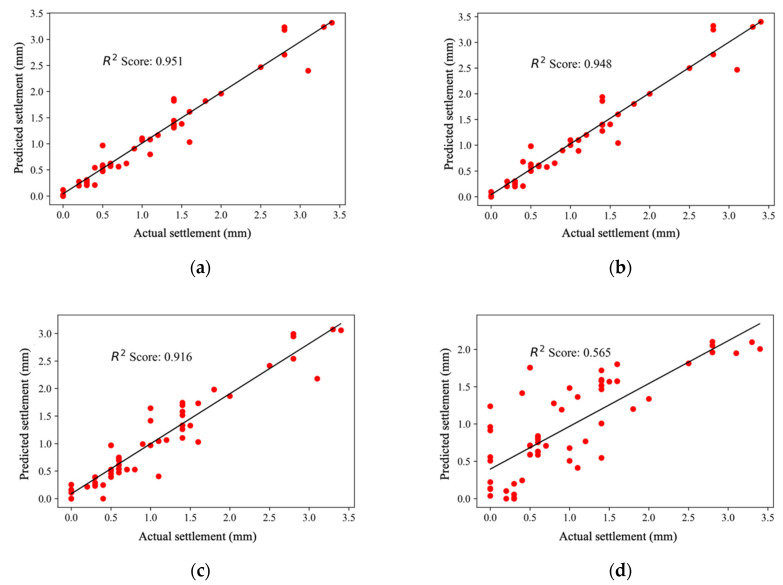
R^2^ comparisons of machine learning models. (**a**) SGM performance; (**b**) SVR performance; (**c**) MLPR performance; (**d**) LR performance.

Table 4 shows the maximum performance of the proposed SGM approach in assessing the liquefaction settlement on the SPT dataset. Figure 9 illustrates the performances of different machine learning models based on R^2^ score. The SGM managed to achieve the best performance with a maximum R^2^ score of 0.951 compared with those of SVR (R^2^ score of 0.948), the MLPR (R^2^ score of 0.916), and LR (R^2^ score of 0.565). The R^2^ score performance of the LR clearly shows that LR alone cannot achieve the best performance. In addition, when LR was applied as a meta-model for the development of the SGM, the model achieved the best performance.

**Figure 10:**
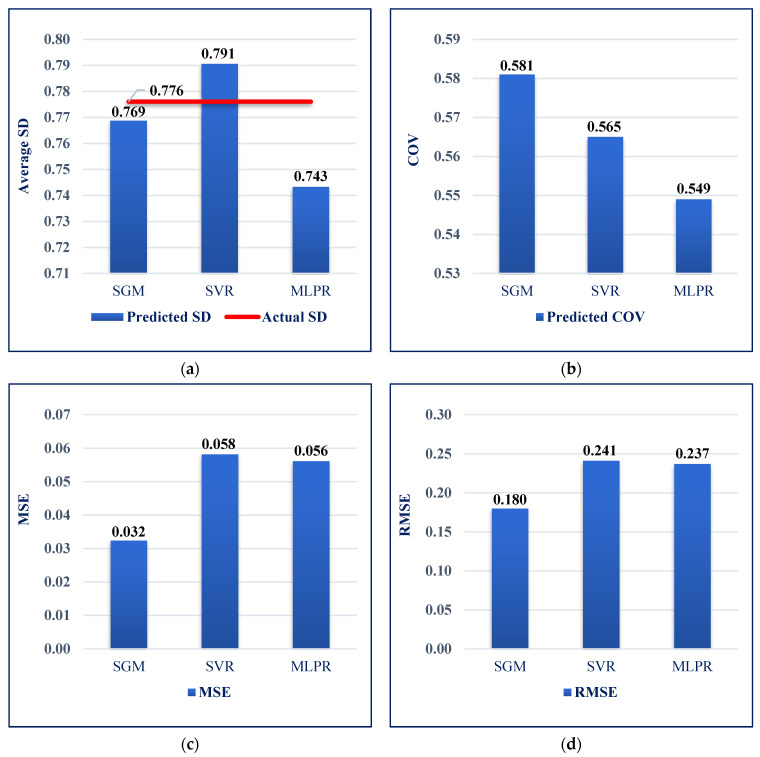
Overall performance comparison of machine learning models. (**a**) Standard deviation comparison; (**b**) covariance comparison; (**c**) mean-square error comparison; (**d**) Root-MSE comparison.

The performance of the SGM was evaluated in terms of SD, COV, MSE, and RMSE metrics (Figure 10). The SD values of the predicted settlement values were very close to the SD values of actual settlement evaluated by the SPT. In addition, the SGM performed better than all other models with regard to the different evaluation parameters. The results also illustrate that apart from SGM, SVR had the second-best performance.

**Figure 11:**
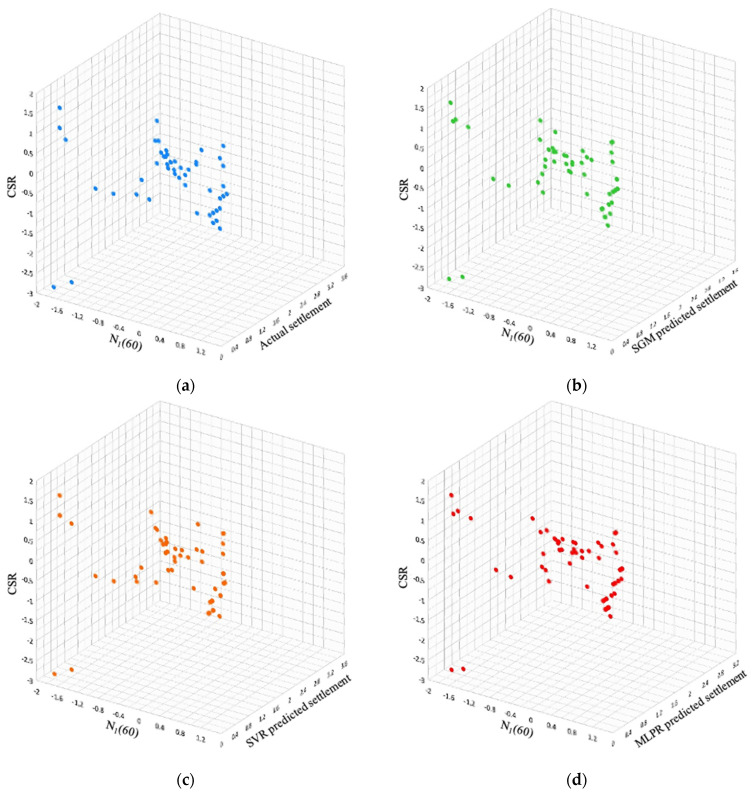
Settlement comparison with CSR and N_1_(60) features of SPT. (**a**) Actual settlement; (**b**) SGM-predicted settlement; (**c**) SVR-predicted settlement; (**d**) MLPR-predicted settlement.

**Figure 12:**
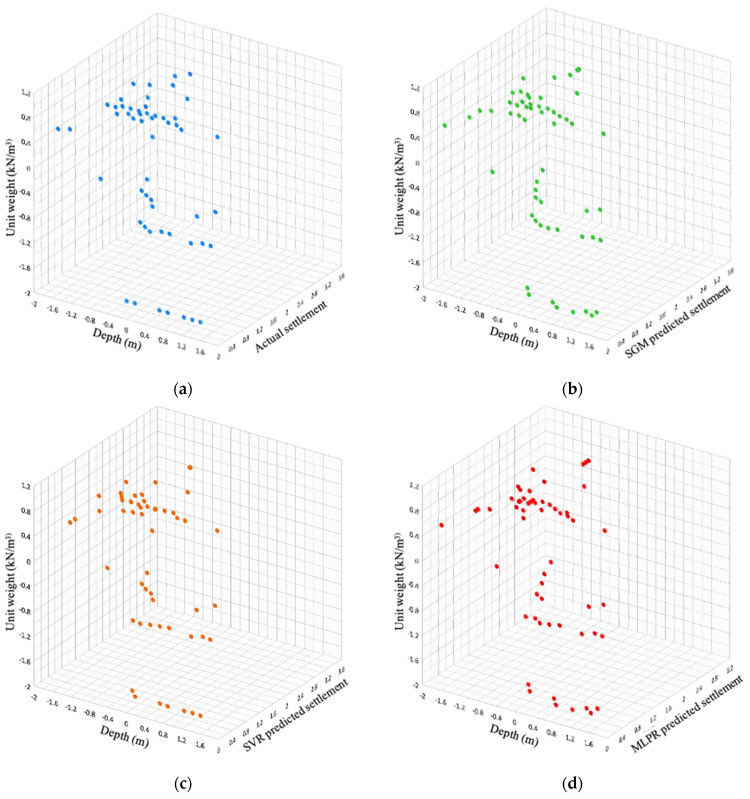
Settlement comparison with unit weight and depth features of SPT. (**a**) Actual settlement; (**b**) SGM-predicted settlement; (**c**) SVR-predicted settlement; (**d**) MLPR-predicted settlement.

The performance of the model with respect to different features available in the SPT dataset was also visualized to provide better insights into the model performance. In Figure 11, the settlement feature is compared with CSR and N1(60). Similarly, the settlement feature is compared with the features unit weight and depth (Figure 12). The visualization shows that the data distribution predicted by the SGM was very close to the actual data distribution.

## 5. Conclusions

This study employed a stacked-generalization-based ensemble approach for liquefication-induced settlement prediction. The experimental dataset was collected from the lab experiments of the SPT. A data augmentation technique using a TGAN was proposed to increase the volume of the SPT dataset. A total of 177 data points were augmented from the original 100 data points to generate a dataset with 277 data points. The complete dataset included five influencing features, namely, depth of the soil layer (depth (m)), CSR, unit weight (kN/m^3^), corrected standard penetration numbers (N_1(60)_), and settlement induced by liquefaction (settlement (mm)). A total of 193 instances of SPT data were used for model training, and 84 cases of the SPT data were reserved for model validation. The proposed SGM approach was integrated with two base models (SVR and MLPR) and a meta-model (LR). Furthermore, the performance of the SGM was validated by all potential assessment metrics, and an enhanced performance was demonstrated compared to other ML models. The SGM managed to enhance the overall performance compared to the existing ensemble approaches.

Moreover, the model parameters of the SVR and MLPR algorithms were fine-tuned efficiently to construct a generalized stacking model. The developed stacking model performed even better on a comparatively modest dataset with mixed features. We inspired a novel configuration of machine learning models to provide robust and accurate results with minimal dataset requirements. The proposed SGM model can overcome the overfitting, low convergence, and poor generalization problems with a small dataset. As a summary of its novelty, SGM is a stacked model built over linear algorithms SVR and MLPR that performs well with a small dataset, overcoming data overfitting, low convergence, and poor generalization. Data imputation is performed on limited iteration to retain the originality of actual data and thus produce reliable performance. Regardless of the substantial performance of the SGM, fine-tuning of the model parameters of the base models and integration of the base models over meta-models remain challenging. The stacking approach demands proper investigation of larger datasets. Additional studies can be conducted on supplementary features along with the existing features of the SPT dataset. The proposed approach can facilitate researchers’ efforts to ascertain the sensitivity of liquefaction-induced settlement over various earthquake datasets.

## Figures and Tables

**Table 1:** Summary statistics of the actual dataset used in this study.

Parameters		Distribution for 100 Actual Standard Penetration Test (SPT) Data Points						
		Mean	Standard Deviation (SD)	25%	50%	75%	Minimum (Min)	Maximum (Max)
Input	Depth (m)	10.50	5.795	5.75	10.5	15.25	1	20
	Unit weight (kN/m^3^)	18.96	1.869	18	20	21	16	21
	N_1(60)_	13.62	8.722	7	11	25	0	25
	CSR	0.314	0.044	0.29	0.32	0.34	0.21	0.39
Output	Settlement (mm)	0.898	0.873	0.3	0.6	1.4	0	3.4

**Table 2:** Augmented data points.

Parameters		Distribution for 177 Augmented SPT Data Points						
		Mean	Std	25%	50%	75%	Min	Max
Input	Depth (m)	10.80	5.321	7	10	15	2	20
	Unit weight (kN/m^3^)	19.31	1.671	18	20	21	16	21
	N_1(60)_	15.25	8.410	10	12	25	1	25
	CSR	0.32	0.030	0.31	0.33	0.34	0.22	0.38
Output	Settlement (mm)	0.98	0.831	0.5	0.6	1.4	0	3.4

**Table 3:** Details of proposed models.

Models	Advantages	Limitations
Support vector regression (SVR)	❖ Easily adaptable ❖ High correlation for nonlinear feature distributions ❖ Not biased by outliers ❖ Can handle the underfitting issues ❖ Learning rate can be optimized easily	❖ Features scaling is mandatory ❖ Difficult to understand ❖ Hyperplane definition for nonlinear feature is complex
Multilayer perceptron regressor (MLPR)	❖ Efficient for nonlinear complex feature distributions ❖ Efficiency increases with large input features ❖ Efficient in learning nonlinear relationships among the features ❖ Works good with continuous values	❖ Low learning will be efficient ❖ Overfitting possibilities in small datasets ❖ Relearning from the features is difficult ❖ Hyperparameter optimization is complex
Linear regression (LR)	❖ Works well irrespective of dataset size ❖ Gives information about the relevance of features ❖ Unambiguous and fast estimation	❖ Complicated assumptions as it assumes the independency between complex features

**Table 4:** Model performance.

Performance Metrics	SGM	SVR	MLPR
R^2^ score	0.951	0.948	0.916
SD	0.769	0.791	0.743
Covariance (COV)	0.581	0.565	0.549
Mean-square error (MSE)	0.032	0.058	0.056
Root-MSE (RMSE)	0.180	0.247	0.237

## Data Availability

Not applicable.
